# Copper Serum Levels in the Hemodialysis Patient Population

**DOI:** 10.3390/medicina60091484

**Published:** 2024-09-11

**Authors:** Guido Gembillo, Luigi Peritore, Vincenzo Labbozzetta, Alfio Edoardo Giuffrida, Antonella Lipari, Eugenia Spallino, Vincenzo Calabrese, Luca Visconti, Domenico Santoro

**Affiliations:** 1 Unit of Nephrology and Dialysis, Department of Clinical and Experimental Medicine, University of Messina, 98125 Messina, Italy; luigiperitore1994@gmail.com (L.P.); vincenzo.labbozzetta@gmail.com (V.L.); alfiogiuffrida91@libero.it (A.E.G.); anto.lipari19@gmail.com (A.L.); eugeniaj@libero.it (E.S.); 2 Unit of Nephrology and Dialysis, Department of Medicine and Surgery, University of Enna “Kore”, 94100 Enna, Italy; v.calabrese@outlook.it; 3 Unit of Nephrology and Dialysis, Ospedali Riuniti Villa Sofia Cervello, University of Palermo, 90146 Palermo, Italy; l.visconti@villasofia.it

**Keywords:** copper, hemodialysis, Cu/zn ratio, chronic kidney disease, zinc, anemia

## Abstract

Copper is an essential element in the diet of mammals, including humans. It plays an important role in the physiological regulation of various enzymes and is consequently involved in several biological processes such as angiogenesis, oxidative stress regulation, neuromodulation, and erythropoiesis. Copper is essential for facilitating the transfer of iron from cells to the bloodstream, which is necessary for proper absorption of dietary iron and the distribution of iron throughout the body. In particular, patients with end-stage renal failure who require renal replacement therapy are at increased risk for disorders of copper metabolism. Many studies on hemodialysis, peritoneal dialysis, and kidney transplant patients have focused on serum copper levels. Some reported mild deficiency, while others reported elevated levels or even toxicity. In some cases, it has been reported that alterations in copper metabolism lead to an increased risk of cardiovascular disease, malnutrition, anemia, or mielopathy. The aim of this review is to evaluate the role of copper in patients undergoing hemodialysis and its potential clinical implications.

## 1. Introduction

Copper (Cu) plays a fundamental role in body homoeostasis and the modulation of various enzymes. Both its deficiency and pathological accumulation can affect normal human health, leading to impaired energy production, abnormalities in glucose and cholesterol metabolism, increased oxidative damage, electrophysiological and contractile problems of the heart, and impaired immune systems; the latter can lead to oxidative stress and tissue damage [1]. 

Both a Cu surplus and a Cu deficiency can have detrimental effects on the body’s homeostasis. Since Cu can act as both a pro- and antioxidant, its influence on oxidation can change depending on consumption [2]. The fact that Cu is a necessary component of copper/zinc superoxide dismutase (SOD) proves the antioxidant properties of copper [3]. Thanks to SOD, all body cells can eliminate free radicals and are thus protected from oxidative stress [4].

While 0.9 mg of Cu is the necessary daily intake, the average diet exceeds this amount by 2.5–5 mg per day [5]. At the cellular and systemic levels, Cu metabolism includes absorption, distribution, sequestration, and excretion. After being absorbed in the proximal small intestine, Cu is transported into the portal circulation by the Cu transporter enzyme ATP7A, where it is loosely bound to albumin. The Cu transporter protein (CTR-1), which is located on the sinusoidal aspect of hepatocytes, is responsible for delivering copper into the cells. Because Cu cannot be found in the cell in either its free or ionic form, it attaches itself to low-molecular-weight proteins called metallochaperones, which transport copper to specific cellular destinations.

Another important aspect for the balance of microelements is maintaining an appropriate ratio between copper and another key element for the body’s homeostasis: zinc (Zn). A balanced ratio of Cu/Zn is necessary for the body’s homeostasis and immune modulation. In addition, the patient’s lipid profile is altered by the loss of zinc and copper, which increases atherogenesis and the associated risk of cardiovascular disease. Interestingly, a reduction in copper can occur when low zinc levels are detected and supplemented with zinc, which can have significant effects. In relation to renal anemia, Cu and Cu/Zinc (Zn) balance are essential for the proper functioning of various regulators of hematopoiesis, including growth hormone, insulin-like growth factor 1, vitamin D receptor, hephaestin, and Cu-Zn superoxide dismutase [6]. By implementing an optimal amount of Cu and Zn, the necessary dosage of erythropoietin can be decreased [7]. Insufficient Cu levels can lead to abnormalities such as myelodysplasia and pancytopenia [8].

Higher concentrations of Cu in the general population have been linked with cardiovascular disease; anorexia [9]; elevated blood concentrations of inflammatory markers [10]; and perhaps, more rapid cognitive decline [11]. Excess dietary Cu can deposit copper in the kidney and lead to nephrotoxicity characterized by proximal tubular necrosis caused by oxidative stress and cellular damage, ultimately leading to a decrease in renal function [12]. However, there is a reciprocal relationship between kidney disease and Cu, as patients with chronic kidney disease (CKD) may also have abnormalities in protein metabolism and reduced renal excretion, which can lead to abnormalities in the homeostasis of circulating Cu levels.

Most such data derive from toxicologic studies of acute or subacute exposure rather than the chronic low-concentration exposure that presumably occurs in patients on renal replacement therapy (RRT). RRT is necessary for patients suffering from end-stage renal disease (ESRD); it can be provided via hemodialysis (HD), peritoneal dialysis (PD), and kidney transplantation. In HD, the patient’s blood is drawn through an arteriovenous fistula or central venous catheter and passed through a dialyzer, where the blood and dialysate exchange fluids and solutes. In this setting, a positive correlation between plasma Cu concentration and the risk of all-cause mortality has been found in HD patients [13].

All these conditions can potentially alter the homeostasis and balance of essential elements, causing unpredictable negative effects and contributing to worsening underlying conditions, comorbidities, and quality of life in our patients.

In our review, we considered English-language studies in human patients on serum, whole blood, and red blood cell data related to copper in HD patients. We excluded studies in which the Cu values could be influenced by zinc supplements, as this microelement is known to influence copper concentration.

**Figure 1:**
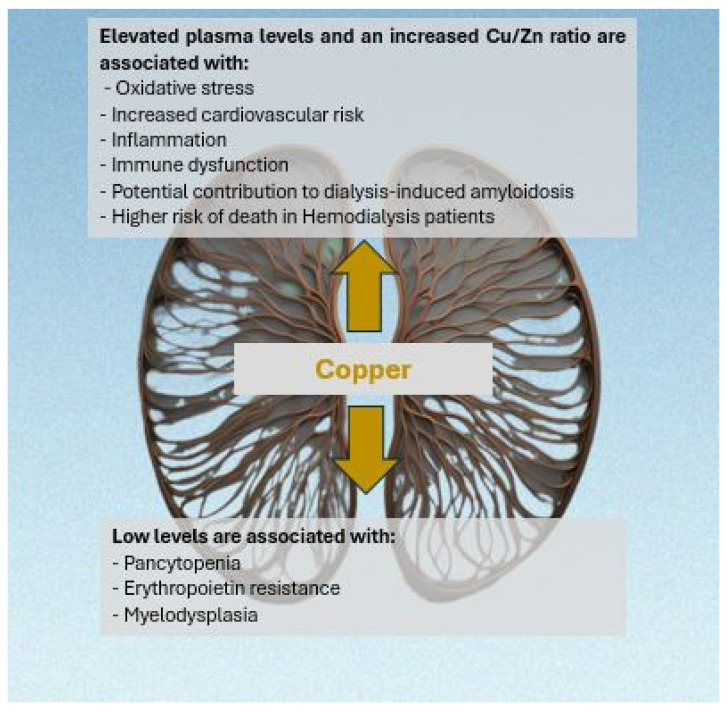
The link between copper, hemodialysis, and continuous renal replacement therapy.

Figure 1 shows the different links between copper and hemodialysis.

## 2. Copper in Hemodialysis

HD is the most common replacement therapy for people suffering from ESRD; worldwide, about 89% of dialysis patients receive HD [14]. A very important problem faced by such patients is the risk of malnutrition due to both iatrogenic and non-iatrogenic factors such as dialysis-related nutrient loss, dialysis-related inflammation, taste changes, loss of appetite, and psychosocial factors [15].

Theoretically, patients undergoing HD are at risk of deficiency of trace elements, including Cu, due to uremia-induced anorexia and restricted diet, as well as for the accumulation of such substances, since even a minimal concentration in the dialysate could create a gradient between it and the blood, with significant differences in the concentrations of microelements in the blood. Many studies have focused on serum Cu status in HD patients. Several studies reported non-significantly lower Cu levels in HD patients compared to control subjects [16,17,18], while some authors reported significantly lower levels than in the control group [19,20].

In their study, Navarro-Alarcon et al. [21] analyzed three different samples that were taken three months apart before each dialysis session. They found that in the first and second samples, the Cu level was higher in HD patients than in healthy controls and that the level was not influenced by the cause of ESRD.

Cu appears to be higher in HD patients with diabetes [22] and in patients with diabetic kidney disease as the main cause of ESRD than in glomerulonephritis, gout, or analgesic or polycystic nephropathy [23]. Ikee et al. [10] reported a positive correlation between serum copper and total cholesterol, LDL cholesterol, and C-reactive protein, while sevelamer appears to be associated with lower copper levels. Recently, Takashi et al. [24] reported that the hypoxia-inducible factor (HIF) inhibitor Roxadustat increases serum copper concentrations in HD patients, while zinc supplementation can decrease serum copper concentrations.

Tonelli et al. [13] analyzed the role of 19 trace elements in HD patients. Of the other trace elements examined in the hypothesis-generating analyzes of the study, only Cu status was clearly associated with a negative outcome; at the *p* < 0.01 level, higher copper concentrations were linked to a higher probability of mortality (Table 1).

Ochi et al. [25] found a significant reduction in the Cu content of the hair of HD patients compared to the control group ((Cu ng/g hair: 12,360 (10,480–17,110) in HD vs 16,385 (13,355–21,225) *p* = 0.0005).

Several authors also investigated the role of Cu in pediatric HD patients. An Iranian cross-sectional study from Tehran University showed no significant difference in Cu serum between a group of HD patients compared to a group of children with end-stage renal disease who were treated conservatively and a control group of healthy subjects [26]. Another study from Mashhad University also found no difference in Cu levels between four groups consisting of children on HD, PD, or conservative treatment, and a control group [27]. Conversely, a retrospective study at Evelina London Children’s Hospital showed that 12 out of 43 patients were Cu-deficient despite taking Cu-containing supplements [28].

Several aspects need to be considered and may partly explain the heterogeneity of the results. Residual renal function, dialyzer membrane and size, type of water used for dialysate preparation, purification technique, pharmacologic therapy, dialysis vintage, and composition of dialysis concentrate can all affect the trace element status of a patient in HD. Another important role played by Cu ions is their possible antimicrobial effect in the HD context. Some authors proposed to develop a new type of HD filter: they polymerized on polyethersulfone a mussel-inspired polydopamine (PDA) to immobilize Cu ions. This filter showed effective antibacterial activity by preventing bacteria from forming biofilms; the amount of Cu released was relatively harmless, potentially reducing the use of antibiotics in ESRD patients [29].

### 2.1. Copper and Anemia in Hemodialysis

Anemia is one of the most important problems faced by HD patients. While the roles of erythropoietin (EPO), hypoxia-inducible factor prolyl hydroxylase inhibitors, iron ions, inflammation, and infection have been well established, Cu deficiency is an often overlooked cause of anemia in this population. Cu plays a fundamental role in erythropoiesis as it is a cofactor of hephaestin, a transmembrane ferroxidase that transports iron from the intestine into the blood and converts iron from divalent to trivalent. Hypocupremia due to excessive Zn intake could be the reason for subsequent anemia and EPO resistance, as shown in the case report of a 56-year-old woman undergoing HD who developed severe anemia with hemoglobin levels between 5 and 7 g/dL despite no signs of bleeding, inflammation, or infection and despite an optimal dose of erythropoietin. Finally, her Zn, Cu, and cerulosplamine levels were determined, which showed a serum Zn level of 236 μg/dL (normal values: 60–120 μg/dL), a serum Cu < 10 μg/dL (normal values: 80–155 μg/dL), and a cerulosplamine level of 3 mg/dL (normal values: 17–54 mg/dL). The woman developed Zn-induced Cu deficiency after taking 220 mg of Zn sulphate daily for a year and using a toothpaste that also caused Zn absorption. She therefore started Cu supplementation therapy and stopped the Zn sulphate. A few weeks later, both her Cu and hemoglobin levels improved and reached normal values [30].

In fact, suboptimal Cu intake could be another cause of anemia in HD patients, especially if neutropenia is also present. Higuchi et al. showed that five patients receiving Cu-free enteral nutrition developed anaemia and neutropenia despite a dose of 9000 UI erythropoietin per week due to very low Cu and ceruloplasmin levels (Cu levels in these patients ranged from 7 to 10 μg/dL and ceruloplasmin levels ranged from 2.7 to 3.6 mg/dL). After three months of Cu supplementation, Cu levels, such as hemoglobin and white blood cells, improved, allowing EPO to be reduced or even discontinued [31].

In a study conducted in Brazil to assess the quality of HD water in a large tertiary hospital and to investigate the relationship between this and clinical abnormalities in patients, the Cu concentration (170 ± 190 μg/dL) was below the maximum level established by Brazilian legislation (100,000 μg/dL): the mixed regression analysis showed that the increase in Cu concentration in the HD water was significant in explaining the altered levels of Cu and was directly related to the anemic status of these patients (r = 2.7478 *p* = 0.0010) [32].

Hypoxia-inducible factor-prolyl hydroxylase inhibitors (HIF-PHIs), used for treating renal anemia, have been found to elevate the levels of Cu in the blood [33]. In their study, Takahashi et al. [24] suggested the use of a combination of HIF-PHI and Zn supplements to prevent a significant increase in serum Cu levels during the administration of HIF-PHI. This approach also allows for safe Zn supplementation without decreasing serum Cu levels in HD patients who are at high risk of Zn deficiency.

### 2.2. Balance of Copper and Zinc in Hemodialysis

Few studies addressed optimal Cu levels in HD patients. In 2020, a descriptive study compared Cu and Zn levels in uremic patients undergoing HD with a control group of healthy people. There was a negative correlation between Zn and Cu in both groups, but the slope was steeper in the HD group, suggesting that an increase in Zn levels reflects a more rapid decrease in Cu levels in these patients. Finally, normal values were calculated for both ions: Zn 41.3–78.3 μg/dL and Cu 66.5–99.5 μg/dL. Moreover, it was found that 7 μg/dL is the highest Zn value that cannot cause Cu deficiency [34].

In 2021, a case report was published on an 89-year-old HD patient who had previously received Zn supplementation and developed Cu deficiency and pancytopenia; the latter improved rapidly after Cu supplementation and discontinuation of Zn. A similar case was previously reported in 2018 in a patient with a history of malabsorption due to gastric bypass [35].

Therefore, the balance between Cu and Zn is very important in Zn supplementation and the concentration of both ions should be checked at least every three months in patients at high risk of Cu/Zn imbalance (Table 2).

If hypocupremia is observed, it should be treated with oral or intravenous Cu in the form of Cu gluconate, Cu chloride, or Cu sulphate. Taking dietary supplements is also an option [6] (Table 3).

Cu deficiency in HD patients can also cause myelopathy, as described in a case report published in 2006 in which a 61-year-old woman developed Cu deficiency caused by Zn supplementation during HD sessions. In this case, neurological symptoms began to improve two weeks after Zn was discontinued and Cu gluconate was introduced [39].

In a cross-sectional study conducted by Batista et al., 33 HD patients with diabetes mellitus (DM), 30 non-diabetic HD patients (NDP), and 20 healthy individuals were examined for the association between end-stage renal disease, diabetic nephropathy, nutritional status, and Zn and Cu levels. Cu intake did not differ between the three groups. In addition, protein intake did not correlate with Zn and Cu intake. Plasma Cu was higher in the diabetic group than in the other two groups (DM: 130.02 ± 6.7 μg/dL vs NDP: 109.7 ± 26.2 μg/dL (*p* < 0.05) vs. control group: 108.0 ± 8.5 μg/dL (*p* < 0.05)). Cu correlated positively with serum glucose (r = 0.44; *p* = 0.0003), HbA1c (r = 0.25; *p* = 0.04), and Kt/V (r = 0.30; *p* = 0.01) and negatively with iron (r = −0.47; *p* = 0.0001), duration of dialysis treatment (r = −0.35; *p* = 0.005), hematocrit (r = −0.33; *p* = 0.009), and adequacy of TSF (r = −0.28; *p* = 0.02). However, in the multivariate analysis, only serum creatinine (β-coefficient = −3.19, CI = −5.12–1.25; *p* = 0.002) and serum glucose (β-coefficient = −0.17, CI = −0.04–0.58; *p* = 0.009) were the influential determinants of Cu. Thus, CKD does not seem to have an influence on Cu concentration because the values were similar in the non-diabetic and healthy groups [22].

As already underlined, HD patients are often malnourished for various reasons. In a Polish study conducted to determine the intake of nutrients, iron, Zn, and Cu in a group of 51 people undergoing HD and compare it with that of a control group of 30 healthy people, it was found that energy, fat, and protein intakes were similar between the two groups, while carbohydrate intake was statistically lower in the patient group compared to the control group (mean g of carbohydrate per day: patient group 182.5 vs. control group: 257.9. *p* < 0.05). Zn, Cu, and iron were lower in the patient group than in the control group. In particular, the average Cu intake in the patient group was 0.7 mg per day, not even 40% of the recommended value [40].

A six-month longitudinal study was conducted at the University of Granada with 48 HD patients and a control group of fifty-two healthy individuals. Thirty-six biochemical indices were measured and correlated with Zn and Cu levels. Blood samples were taken at baseline and after 3 and 6 months. The mean serum levels of Zn and Cu were 63.5, 70.1, and 80 μg/dL and 113.6, 128.8, and 118.8 μg/dL, respectively. For Cu, no statistically significant differences were found among the three samples, but a significant difference was observed compared to the control group for serum samples collected in period 1 and period 2 (113.6 ± 4.5 μg/dL in period 1 vs. 128.8 ± 4.7 μg/dL in period 2 from HP compared to 108.3 ± 4.1 μg/dL in the control group. *p* < 0.05). Measured Cu levels were not statistically affected by disease ethology, while concentrations were higher in males than females (*p* < 0.01). In all regression analyses, there were only two significant linear relationships (*p* < 0.05) for Cu with total cholesterol and glutamic pyruvic transaminase concentration. Finally, serum Cu levels were significantly altered in patients with secondary concomitant disease such as diabetes, cardiopathies, infections, hyperparathyroidism, and hepatopathies (*p* < 0.01). In particular, patients with diabetes or hepatopathies had significantly higher Cu levels than the control groups [21].

### 2.3. Copper/Zinc Ratio in Hemodialysis

Elevated plasma levels of Cu and an increased Cu/Zn ratio are associated with nuclear abnormalities, oxidative stress, inflammation, and immune dysfunction [41]. Chih-Hung Guo found a positive correlation between the Cu/Zn ratio and hs-CRP (r = 0.354, *p* = 0.03), TNF-alfa (r = 0.359, *p* = 0.02), and IL -1beta (r = 0.218, *p* = 0.18) in a study comparing 40 HD patients receiving 78 mg of Zn gluconate with a group of patients not taking Zn and with a group of healthy people. In addition, patients in the Zn group had a higher CD4/CD8 ratio and a higher proportion of CD19 than the control group. Thus, the administration of low doses of Zn reduced the high plasma Cu/Zn ratios in chronic HD patients and helped to reduce inflammation and positively influence immune function [42].

An increased Cu/Zn ratio in HD patients also means an increased cardiovascular risk. In a two-year longitudinal study conducted at the University of Granada, 116 people with end-stage renal disease who had been hemodialyzed for at least six months were studied and compared with a group of 50 healthy people. The Cu/Zn ratio was significantly higher in the group of patients compared to the control group (151.1 ± 59.6 μg/dL vs. 91.4 ± 20.7 μg/dL, *p* < 0.01). Serum Cu concentration was higher in patients with diabetic nephropathy (μg/dL: 130.9 ± 21.6) than in patients with glomerulonephritis (99.2 ± 25.4 μg/dL), with gout (100 ± 12.7 μg/dL), taking analgesics (103.2 ± 21.6 μg/dL), or with polycystic nephropathy (103.8 ± 11.7 μg/dL) (*p* < 0.05). Cu levels were significantly higher in patients receiving antihypercalcemics (104.7 ± 27 μg/dL vs. 94.1 ± 28.1 μg/dL) and in those taking infarct drugs (111 ± 25.2 μg/dL vs. 99.9 ± 27.1 μg/dL) than in patients not taking these drugs, while they were lower in patients receiving diuretics (94.8 ± 25.7 μg/dL vs. 104.2 ± 27 μg/dL). Finally, a positive linear correlation was found between Cu/Zn levels and total cholesterol levels (r = 0.111 *p* = 0.022) and LDL cholesterol levels (r = 0.111 *p* = 0.049); an increase in these levels was associated with an increased cardiovascular risk [23].

### 2.4. Copper and Hemodialysis-Related Amyloidosis

Human β2-microglobulin is an amyloidogenic protein that tends to accumulate in patients who require intermittent HD for prolonged periods. Clinically, this accumulation leads to dialysis-related amyloidosis syndrome, causing joint pain, bone erosion, and nerve compression symptoms. Several experiments provided data showing that Cu ions play a fundamental role in promoting the organization of such proteins into dimers and tetramers that precede amyloid formation, and that this process is enhanced by urea levels similar to those seen in HD patients [43]. Calabrese et al. [44] also speculated that the transient aggregation of β2-macroglobulin with Cu ions at the interface of the dialysis membrane could be the cause of dialysis-induced amyloidosis. Furthermore, Cu ions show a high affinity to other amyloidogenic proteins such as β-amyloid peptides, Ig light chains, the prion proteins, and α-sinuclein [45,46]. Finally, the incidence of amyloidosis has decreased significantly since the elimination of Cu from the dialysis membrane [47].

## 3. Conclusions

The changes in serum trace element concentrations suggest that control of these nutrients is critical for ESRD patients requiring RRT. Experimental models in vitro as well as animal and human studies have shown that Cu may play an important role in mechanisms related to the progression of CKD and its complications. These findings have also been investigated in the various renal function replacement techniques. Evidence for the association between Cu and important dialysis-related outcomes remains limited and the results are conflicting. High Cu levels appear to be related to higher cardiovascular risk and mortality in HD patients, while low levels are associated with persistent anemia and resistance to therapy. Unfortunately, there are significant differences in the various methods and timing of copper sampling, which is a major limitation in the available studies.

Further studies are needed to adequately investigate the role of Cu in patients on renal replacement therapy, and the results available in the literature remain controversial.

## Figures and Tables

**Table 1:** Caption. Different studies involving serum copper levels in hemodialysis patients.

Authors	Years	Patients	Serum Copper Levels	Note
Emenaker et al. [19]	1996	23 HD patients (gender not specified) Mean age 50 (28–77) Dialysis vintage not specified Membrane of cellulose acetate, saponified cellulose esters, or polysulfone-based membranes	134.6 ± 27.1 μg/dL	Significantly lower levels than control
Zima et al. [16]	1998	36 HD patients (19 males, 17 females) Mean age 57 (24–77) Dialysis vintage not specified Low-flux dialyzers	69 ± 3 μg/dL	No significant difference in serum copper levels between HD patients, PD patients, and control group
Batista et al. [22]	2005	63 HD patients (31 males/32 females) 33 with DM2, 30 without DM2 Dialysis vintage 29.0 ± 27.4 months in DM group Dialysis vintage 43.3 ± 33.5 months in non-DM group Acetate cellulose membrane	Diabetic: 130.02 ± 36.7 μg/dL (pre-HD); Non-diabetic: 109.7 ± 26.2 μg/dL (pre-HD)	Increased plasma copper in diabetic group (*p* < 0.05)
Alarcon et al. [21]	2005	48 HD patients (32 males, 16 females) Mean age: 50.1 ± 11.2 Dialysis vintage not specified Membrane not specified	Three samples were taken before each dialysis session at three different times, 3 months apart each First sampling: 113.6 ± 4.5 μg/dL Second sampling: 128.8 ± 4.7 μg/dL Third sampling: 118.8 ± 5 μg/dL	No statistically significant differences among the three blood samples. *p* > 0.05 In first and second samples, copper levels were higher in HD patients than in healthy controls; *p* < 0.05 Copper levels were not influenced by cause of ESRD
Hsieh et al. [17]	2005	77 HD patients Mean age not specified Dialysis vintage not specified Membrane not specified	94,190 ± 16,970 μg/dL	Cu concentration was statistically the same between the hemodialysis and control groups
Koca et al. [18]	2010	110 HD patients (46 males, 54 females) Mean age 54 ± 14 Dialysis vintage: 0–2 years n = 31; 3–5 years n = 40; 6–8 years n = 27; 9–11 years (n = 13) Polysulfone-based membranes	0–2 years of HD = 111.1 ± 28.6 μg/dL (pre-HD) 3–5 years of HD = 107.6 ± 21.4 μg/dL (pre-HD) 6–8 years of HD = 106.1 ± 33.3 μg/dL (pre-HD) 9–11 years of HD = 129.8 ± 34.7 μg/dL (pre-HD)	No statistically significant differences between control and HD groups and no significant difference between different dialysis age groups
Ikee et al. [10]	2012	48 HD patients (28 males/20 females) Age 71 ± 10 Dialysis vintage 84 ± 72 months Polysulfone membranes in all patients	94 μg/dL (pre-HD)	Positive correlations between serum copper and total cholesterol, LDL-cholesterol, and C-reactive protein and negative correlations with sevelamer dose
Eleftheriadis et al. [20]	2014	34 HD patients (23 males, 11 females) Age 59.5 ± 13.6 Dialysis vintage 58.3 ± 40.2 (months) Polysulfone low-flux dialyzers	64.77 μg/dL	Serum copper in HD patients was reduced compared to controls; *p* < 0.001
Reina de la Torre et al. [23]	2014	116 HD patients (51 male, 65 females) Age 74.6 ± 11.44 years Dialysis vintage at least 6 months, not further specified Low-flux polysulfone membranes	102.2 ± 26.6 μg/dL (pre-HD)	Serum Cu levels did not significantly differ between HD patients and controls (*p* = 0.059) Copper was higher in those with diabetic nephropathy compared to glomerulonephritis, gout, or analgesic or polycystic nephropathy
Tonelli et al. [13]	2018	1278 HD patients (784 males/494 females) Age 62 ± 10 Dialysis vintage not specified High-flux polysulfone membranes	Not specified	Higher copper concentrations were associated with higher risk of death (odds ratio, 1.07 per decile; 99.2% coincidence interval, 1.00 to 1.15)
Takahashi [6]	2023	40 HD patients (20 males/20 females) Age 75 ± 11 Dialysis vintage 84 ± 60 Membranes not specified	173 ± 27.9 (μg/dL) (no zinc no Roxadustat) 147.2 ± 35.1 (μg/dL) (zinc, no Roxadustat) 291.4 ± 46.4 (μg/dL) (no zinc, post Roxadustat) 182.1 ± 59.9 (μg/dL) (zinc, post Roxadustat)	Roxadustat increases the serum copper concentrations of patients, and zinc supplementation can reduce serum copper concentrations

**Table 2:** Recommended dietary allowances (RDAs) for copper and zinc [36].

Age	Cu RDAs for Male (mcg)	Cu RDAs for Female (mcg)	Zn RDAs for Male (mg)	Zn RDAs for Female (mg)
Birth to 6 months	200	200	2	2
7–12 months	220	220	3	3
1–3 yrs	340	340	3	3
4–8 yrs	440	440	5	5
9–13 yrs	700	700	8	8
14–18 yrs	890	890	11	9
19+ yrs	900	900	11	8

Cu: copper; Zn: zinc; mcg: microgram, mg: milligram, yrs: years.

**Table 3:** Recommended values for copper, zinc, and ceruloplasmin concentrations for the general population [37,38].

Microelements	Serum Levels
Copper	10–25 mcmol/L (63.5–158.9 mcg/dL)
Zinc	12 to 18 mcmol/L (80 to 120 mcg/dL)
Ceruloplasmin	180–400 mg/L

Micromoles per liter (mcmol/L), mcg: microgram; mg: milligram.

## Data Availability

Not applicable.

## Abbreviations

AKI	Acute Kidney Injury
CKD	Chronic Kidney Disease
CRRT	Continuous Renal Replacement Therapy
Cu	Copper
CuD	Copper-Deficient Diet
CuS	Copper-Supplemented Diet
DM	Diabetes Mellitus
EPO	Erythropoietin
ESRD	End-Stage Renal Disease
HD	Hemodialysis
hs-CRP	High-Sensitivity C Reactive Protein
HVHF	High-Volume Hemofiltration
ICU	Intensive Care Unit
IQR	Interquartile Range
IL-1 beta	Interleukin-1 Beta
LDL	Low-Density Lipoprotein
pCU	Plasma Copper
PD	Peritoneal Dialysis
RBC	Red Blood Cells
RRT	Renal Replacement Therapy
ROS	Reactive Oxygen Species
SOD	Superoxide Dismutase
TSF	Transferrin
Zn	Zinc
